# Greek Remdesivir Cohort (GREC) Study: Effectiveness of Antiviral Drug Remdesivir in Hospitalized Patients with COVID-19 Pneumonia

**DOI:** 10.3390/microorganisms10101949

**Published:** 2022-09-30

**Authors:** Vasilis Petrakis, Vasiliki Rapti, Karolina Akinosoglou, Constantinos Bonelis, Kalomoira Athanasiou, Vasiliki Dimakopoulou, Nikolaos K. Syrigos, Nikolaos Spernovasilis, Grigoris Trypsianis, Markos Marangos, Charalambos Gogos, Dimitrios Papazoglou, Periklis Panagopoulos, Garyfallia Poulakou

**Affiliations:** 1 2nd Department of Internal Medicine, Democritus University of Thrace, University General Hospital Alexandroupolis, 68100 Alexadroupolis, Greece; 2 3rd Department of Internal Medicine, National and Kapodistrian University of Athens, Sotiria General Hospital, 11527 Athens, Greece; 3 Department of Internal Medicine and Infectious Diseases, Medical School, University of Patras, 26504 Patras, Greece; 4 Department of Internal Medicine, Medical School, University of Patras, 26504 Patras, Greece; 5 Harvard School of Public Health, Boston, MA 02115, USA; 6 School of Medicine, University of Crete, 71500 Heraklion, Greece; 7 Department of Infectious Diseases, German Oncology Center, Limassol 4108, Cyprus; 8 Department of Medical Statistics, Medical School, Democritus University of Thrace, 68100 Alexandroupolis, Greece

**Keywords:** remdesivir, COVID-19, SARS-CoV-2, pandemic, retrospective study, intubation, discharge

## Abstract

In several randomized studies, remdesivir (RDV) has been reported to shorten the recovery period and improve clinical outcomes in COVID-19 patients, and thus, it is recommended as a standard of care. Nevertheless, controversial reports have been published. The aim of the present study is to evaluate the effectiveness of remdesivir in hospitalized patients with COVID-19 pneumonia at three Greek University Departments of Infectious Diseases with homogenous treatment protocols. From September 2020 to February 2021, we retrospectively analyzed adults hospitalized with confirmed SARS-CoV-2 infection and radiological findings of pneumonia, who received remdesivir once daily for five days. Exploratory end points were duration of hospitalization, time of intubation, and death. Overall, 551 patients were included in the study. The optimal cutoff point for the number of days needed after symptom initiation for drug administration associated with better clinical outcome was 7 days. Higher odds for discharge and lower for intubation were observed in patients with treatment initiation ≤7 days (*p* = 0.052 and *p* = 0.019, retrospectively) regardless of gender (*p* = 0.537), hypertension (*p* = 0.096), dyslipidemia (*p* = 0.221), diabetes mellitus (*p* = 0.306), and usage of immunomodulators (*p* = 0.408). Our study has demonstrated beneficial effects of early treatment with remdesivir (≤7 days from symptom onset) on rates of intubation and probability of discharge.

## 1. Introduction

Severe acute respiratory syndrome coronavirus 2 (SARS-CoV-2) responsible for the coronavirus disease 2019 (COVID-19) induced an initial outbreak, in December 2019, in Wuhan, China, and rapidly led to a pandemic [1,2]. Effective antiviral treatments are vital in order to reduce mortality rates and hospitalizations and to protect vulnerable populations.

Remdesivir (GS-5734), a monophosphoramidate nucleoside prodrug with known antiviral activity against SARS-CoV and the Middle East respiratory syndrome coronavirus (MERS-CoV), was evaluated for its efficacy against SARS-CoV-2 early during the COVID-19 pandemic [3,4]. Upon entry into target cells, remdesivir undergoes metabolic conversion into its active form, remdesivir triphosphate (remdesivir-TP), which competes with adenosine triphosphate (ATP) for incorporation into the nascent RNA strand and acts as substrate for RNA dependent RNA polymerase (RdRP), thus, inhibiting viral replication machinery [5,6,7]. Remdesivir-derived RdRP inhibition is mainly due to delayed chain termination resulting from (i) misintegration of nucleoside triphosphate (NTP) into replicating RNA by RdRp, (ii) prevention of further chain elongation after NTP plus three additional nucleosides, and (iii) premature termination of RNA synthesis [7].

According to the results of the Adaptive COVID-19 Treatment Trial (ACTT-1) (a double-blind, randomized, placebo-controlled trial) remdesivir was associated with a shorter period to recovery [8]. In contrast to the initial jeopardy, the final results of the WHO Solidarity randomised trial and the Canadian Treatments for COVID-19 (CATCO) trial have also documented beneficial roles of remdesivir in survival rates and progression to ventilation in hospitalized patients with COVID-19 pneumonia [9,10]. However, there is still controversy in the existing guidelines on the use of remdesivir in hospitalized patients with COVID-19 [11,12,13].

The effectiveness of remdesivir is associated with the time of treatment initiation. Viremia at admission is a predictor of mortality and poor viral control contributes to disease severity [14]. Studies have shown that the duration of viremia is approximately 10 days, and thus during this period, antiviral treatment should be initiated [15]. Initiation of remdesivir prior to or simultaneously with dexamethasone has been associated with significantly shorter time to clinical improvement and lower mortality risk [16]. Patients with moderate COVID-19 treated with remdesivir for up to 5 days had significantly higher odds of better clinical outcome [17]. Based on these data, the PINETREE clinical trial in non-hospitalized patients at high risk for disease progression reported that a 3-day course of remdesivir resulted in an 87% lower risk of hospitalization or death [18].

The aim of the present retrospective study was to evaluate the effectiveness and the optimal time initiation of remdesivir in hospitalized patients with COVID-19 pneumonia at three University Departments of Infectious Diseases (Athens, Alexandroupolis, and Patra) during the period after the European Medicines Agency (EMA) approval until initiation of vaccination against SARS-CoV-2 (September 2020–February 2021) [19]. We also sought to evaluate our practice by comparing it to the international and national recommendations.

## 2. Materials and Methods

This was a retrospective study conducted at three University Departments of Infectious Diseases (Athens, Alexandroupolis, and Patra). Data from routine care patient charts during the period September 2020 to 28 February 2021 were retrospectively analyzed. The study was carried out in accordance with the Helsinki Declaration of Human Rights.

Adults with confirmed SARS-CoV-2 infection and radiological findings of pneumonia were included in the study. The patients were treated with remdesivir intravenously for five days (200 mg on Day 1, 100 mg on Days 2, 3, 4, and 5). The analysis included: age, gender, comorbidities, usage of immunomodulatory agents, and number of days after symptom onset for first dosage of remdesivir. Exploratory end points were duration of hospitalization, time of intubation, and death.

The statistical analysis of the data was performed using the IBM Statistical Package for the Social Sciences (SPSS), version 19.0 (IBM Corp., Armonk, NY, USA). The normality of quantitative variables was tested using the Kolmogorov–Smirnov test. Normally distributed quantitative variables were expressed as the mean ± standard deviation (SD), while non-normally distributed quantitative variables were expressed as the median value and range. Qualitative variables were expressed as absolute and relative (%) frequencies. A receiver operating characteristic curve (ROC) analysis was performed in order to evaluate the optimal cutoff point for the number of days from the symptom onset until initiation of remdesivir associated with better clinical outcome. Student’s *t*-test, Mann–Whithey U test, and chi-square test were used to determine differences in demographic and clinical characteristics of patients. All tests were two tailed and statistical significance was considered for *p*-values < 0.05.

## 3. Results

**Figure 1:**
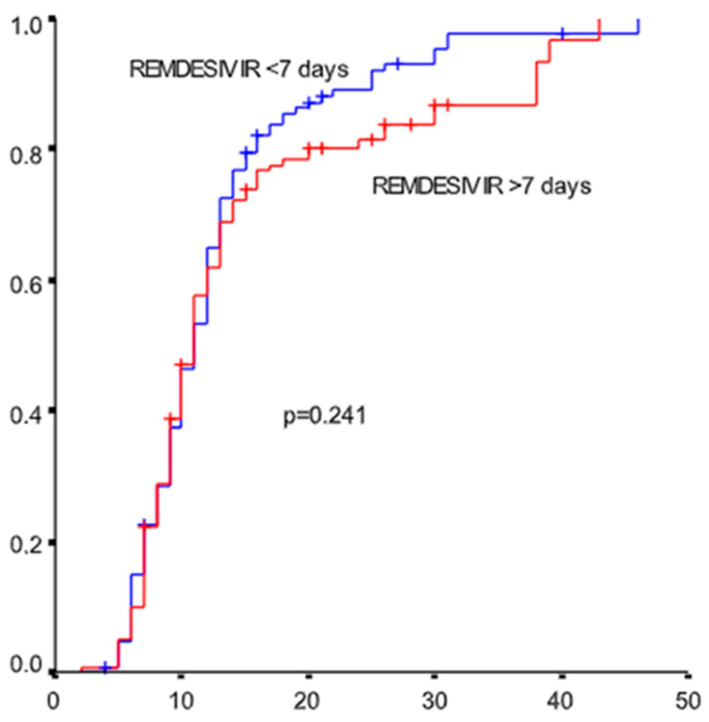
Seven days after symptom onset as the optimal time of remdesivir initiation (ROC analysis).

Data from 551 patients (335 males, 60.8%) with a median age of 59.97 ± 14.538 years were analyzed. Comorbidities were present in 57.5% of individuals; the most common comorbidities were hypertension (40.1%), dyslipidemia (27.6%), diabetes mellitus (20.3%), and coronary disease (11.3%). One comorbidity was documented in 27.6% of patients, while 30% of patients had two or more comorbidities. Immunomodulatory agents were added in the treatment of 157 patients (28.5%). The optimal cutoff point for the number of days needed after symptom onset for drug administration associated with better clinical outcome was 7 days (Figure 1). The ROC analysis showed that the number of days with symptoms was a significant predictor for progression to ventilation with sensitivity 50% and specificity 65%. The results of the study are shown in Table 1.

The percentage of patients who were discharged after COVID-19 hospitalization was 90% (496 individuals). A higher percentage of discharge was reported for patients with treatment initiation ≤7 days (91.2%, 320 out of 351 patients) as compared with those treated with remdesivir more than seven days after symptom onset (88.0%, 176 out of 200) (*p* = 0.233). Probability of recovery was 67% higher in patents with treatment initiation ≤7 days (adjusted odds ratio (aOR) = 1.67, 95% CI 0.91–3.08, *p* = 0.099) after adjustment for gender, age, comorbidities, and usage of immunomodulatory agents. The likelihood of recovery was also higher in females (aOR = 1.96, 95% CI 1.01–3.77, *p* = 0.045) and 8% lower for one year increase in age (aOR = 0.92, 95% CI 0.89–0.95, *p* < 0.001). The median duration of hospital stay was 9 days for patients who recovered and 20 days for those who died. The median time for discharge had no significant difference regarding the time of treatment initiation, ≤7 or >7 days (9 days vs. 10 days, *p* = 0.084 in all patients; 9 days vs. 9 days, *p* = 0.370 in patients who recovered; 16 days vs. 25 days, *p* = 0.150 in patients who died).

Intubation was needed for 60 patients (10.9%). Higher odds of intubation was observed when treatment with remdesivir was started more than 7 days after symptom onset (aOR = 1.88, 95% CI 1.07–3.31, *p* = 0.028). The probability of intubation was higher in males (aOR = 1.82, 95% CI 0.98–3.37, *p* = 0.056) and in patients who were treated with immunomodulatory agents (aOR = 2.04, 95% CI 1.14–3.65, *p* = 0.016). Death occurred in 55 individuals (10%). Higher odds of death was reported in males (aOR = 1.96, 95% CI 1.01–3.77, *p* = 0.045). The likelihood of death was 67% higher among patients in whom treatment with remdesivir was initiated more than 7 days after symptom onset (aOR = 1.67, 95% CI 0.91–3.08, *p* = 0.099). The median duration of hospitalization in patients who recovered was significantly lower than in patients who died (9 days (IQR = 6–12 days) vs. 20 days (IQR = 10.75–30 days), *p* < 0.001). The ROC analysis showed that the optimal cutoff point for survival was 14.5 days of hospital stay with sensitivity 70.4% and specificity 84.7% (area under the curve (AUC) = 0.793, *p* < 0.001).

## 4. Discussion

This is the first study to report experience with remdesivir use in Greece during the first two pandemic waves. In this retrospective study, a 5-day course of remdesivir initiated within 7 days after symptom onset was associated with a shorter time to recovery, a shorter length of hospital stay, and lower mortality rate. Treatment with remdesivir may have prevented the progression to more severe respiratory disease, as shown by the lower likelihood of intubation eventually in patients receiving low-flow oxygen. 

Our data were in line with the ACTT-1 randomixed clinical trial, which in a total of 1062 patients showed that patients treated with remdesivir had a median recovery time of 10 days (95% CI 9–11) as compared with 15 days (95% CI 13–18) among those who received placebo (rate ratio for recovery, 1.29 and 95% CI 1.12–1.49) [8]. The mortality was 6.7% with remdesivir and 11.9% with placebo by Day 15 and 11.4% with remdesivir and 15.2% with placebo by Day 29 (hazard ratio (HR), 0.73 and 95% CI 0.52–1.03) [8]. Nevertheless, some conflicting results for the clinical efficacy of remdesivir were reported afterwards. The DisCoVeRy, a phase 3, open-label, adaptive, multicentre, randomized, controlled trial conducted in 48 sites in Europe evaluating the clinical efficacy of remdesivir plus standard of care as compared with standard of care alone in hospitalized patients with oxygen or ventilator support, showed no association of remdesivir with a better clinical outcome at Days 15 and 29 nor with a shorter time for viral clearance [20]. Similar findings were reported in the interim results of the WHO Solidarity trial [21]. However, further accumulating data from clinical trials and real life argue clearly for a beneficial role of remdesivir in the treatment of COVID-19 pneumonia, showing an association with clinical improvement and increased chance of recovery along with reduced disease progression and mortality [22,23,24,25]. Remdesivir was associated with significantly higher rates of recovery at Day 14 (aOR = 2.03, 95% CI 1.34–3.08, *p* < 0.001), increasing chance of hospital discharge but also decreasing all-cause mortality in a real-world retrospective cohort including Belgium, Germany, and Hong Kong during the first pandemic wave [23]. However, the benefits did not extend to every subgroup, mainly driven by patients with low-flow oxygen [23].Time to clinical improvement was shorter with the use of remdesivir in a retrospective cohort involving several sites in the USA [22]. RDV was associated with a statistically significant increase in the likelihood of clinical improvement, a benefit also driven primarily by patients on no or low-flow oxygen [22]. RDV was associated with a 22% statistically significant faster recovery vs. a control between hospital Day 9 and Day 28; however, the chance of recovery varied with time in a British cohort [26]. A Korean cohort revealed significantly greater viral load reduction in the upper respiratory tract with RDV vs. supportive care, while progression to MV by Day 28 was significantly lower and MV duration significantly shorter with RDV vs. supportive care [25]. The benefits of early treatment have been previously noted in various settings [26,27,28]. Earlier remdesivir administration lowered all-cause mortality in a U.S. retrospective cohort involving 190,529 patients with mild to severe COVID-19 pneumonia [27]. Spanish data confirmed previous observations that RDV was associated with a survival benefit that increased with shorter duration of symptoms before admission [28,29]. Pre-admission symptom durations of 4—6 days and ≤3 days were associated with a 1.5- and 2.5-fold increases in the 30-day mortality rate, respectively, as compared with >6 days, while RDV treatment was independently associated with a lower mortality rate (OR 0.38, 95% CI 0.22, 0.67) [28,29]. Data were similar to those of Asian and U.S. cohorts, reporting early RDV treatment was also associated with a significantly shorter length of hospital stay (difference, 2.56 days; 95% CI −4.86, −0.26; *p* = 0.029) [16,30]. This is not surprising taking into consideration its mechanism of action, underlining its use early in the course of the disease, when viral replication is dominating, rather than later stages of systemic inflammatory response [3,4,14].

It seems that access to early treatment is an important component of successful management of COVID-19. Early reports of high mortality were undoubtedly associated with overwhelmed health systems and patients waiting at home for prolonged periods before hospital admission [31]. The centers participating in the analysis have never experienced patient overflows due to adequate local population/hospital bed ratios and a lack of a bottleneck effect on admission decisions. The areas covered by the two regional University Hospitals of Alexandroupolis and Patra did not experience any bed shortage during the first two pandemic waves, whereas, for the third center serving Athens Metropolitan area, the method of rotating service ensured that there was adequate bed availability [32,33]. Despite the on-off pattern of other Greek centers according to the national policy for expanding COVID-19 beds according to the flow of the pandemic, it is noteworthy that all three participating centers of this study were implicated in the COVID-19 response without interruption, from the very first day of the pandemic, a fact that might have contributed to the early adoption of evolving beneficial supportive treatments to patients with COVID-19.

Based on the above, we believe that this retrospective study allowed us to shed light on early treatment with remdesivir in patients with COVID-19 pneumonia under minimal stress of the healthcare system, and thus, elucidate a clear beneficial effect. We strongly believe that analysis of local data is very important in decisions affecting public health and can guide local authorities to adopt strategies with high probabilities of favorable cost/benefit ratios. It is worth mentioning that interpretation of the literature has produced significant variations in the recommendation of treatment with remdesivir in patients with mild COVID-19 pneumonia [11,12,13].

Our National Therapeutic Algorithm for the treatment of inpatients with COVID-19, in the most recent version of 14 February 2022, has adopted a strategy similar to the NIH guidelines, recommending remdesivir in all inpatients without need of oxygen if they are at increased risk for severe disease, in all inpatients with low-flow oxygen need, and in all patients on non-invasive ventilation or high-flow nasal canula (in combination with dexamethasone +/− immunomodulators) [34]. Finally, remdesivir (in combination with dexamethasone +/− immunomodulators) is recommended for mechanically ventilated patients if it was already started before intubation and only for a total of five days. In all cases, remdesivir is recommended only within the first seven days from COVID-19 symptom onset [34]. We believe that the results of the current study clearly support the national algorithm and it is the first study that provides data in support of the National COVID-19 treatment strategy with the use of real-world data.

The limitations of the study include its retrospective character, the small number of patients, and the absence of a control group with another therapeutic regimen

## 5. Conclusions

In conclusion, based on published data, remdesivir was associated with better clinical outcome and shorter period of recovery. Our study has demonstrated the significance of early treatment with remdesivir (≤7 days from COVID19 symptom onset) in order to achieve reduced rates of intubation and mortality and higher probability of discharge. Our data fully support the decisions that were taken by the National Committee on the response to COVID-19 pandemic and the therapeutic algorithm that was adopted.

## Figures and Tables

**Table 1:** Characteristics and clinical outcome of patients based on time of remdesivir initiation.

	Total	Initiation ≤7 Days	Initiation >7 Days	*p* Value
**Male gender, n (%)**	335 (60.8)	210 (59.8)	125 (62.5)	0.537
**Age**	59.97 ± 14.38	60.40 ± 14.93	59.23 ± 13.34	0.356
**Immunomodulators**	157 (28.5)	83 (23.6)	74 (37.0)	0.001
**Comorbidities**				0.145
Hypertension	221 (40.1)	150 (42.7)	71 (35.5)	0.096
Diabetes	112 (20.3)	76 (21.7)	36 (18.0)	0.306
Dyslipidemia	152 (27.6)	103 (29.3)	49 (24.5)	0.221
CVD	62 (11.3)	44 (12.5)	18 (9.0)	0.207
None	234 (42.5)	141 (40.2)	93 (46.5)	
One	152 (27.6)	95 (27.1)	57 (28.5)	
Two or more	165 (29.9)	115 (32.8)	50 (25.0)	
**Recovery**	496 (90.0)	320 (91.2)	176 (88.0)	0.233
**Time to recovery**	9 (6–13)	9 (6–12)	9 (6–12.75)	0.370
**Intubation**	60 (10.9)	30 (8.5)	30 (15.0)	0.019
**Time to intubation**	10.5 (3.5–14.75)	8.5 (3–12.5)	12 (8.25–17.25)	0.067
**Mortality at day 14th**	16 (3.7)	10 (3.6)	6 (3.8)	0.884
**Mortality (overall)**	55 (10.0)	31 (8.8)	24 (12.0)	0.233

## Data Availability

Not applicable.

## References

[1] Lu R., Zhao X., Li J., Niu P., Yang B., Wu H., Wang W., Song H., Huang B., Zhu N. (2020). Genomic characterisation and epidemiology of 2019 novel coronavirus: Implications for virus origins and receptor binding. Lancet.

[2] World Health Organization. https://www.who.int/emergencies/diseases/novel-coronavirus-2019/situation-reports.

[3] Agostini M.L., Andres E.L., Sims A.C., Graham R.L., Sheahan T.P., Lu X., Smith E.C., Case J.B., Feng J.Y., Jordan R. (2018). Coronavirus Susceptibility to the Antiviral Remdesivir (GS-5734) Is Mediated by the Viral Polymerase and the Proofreading Exoribonuclease. mBio.

[4] Gordon C.J., Tchesnokov E.P., Woolner E., Perry J.K., Feng J.Y., Porter D.P., Götte M. (2020). Remdesivir is a direct-acting antiviral that inhibits RNA-dependent RNA polymerase from severe acute respiratory syndrome coronavirus 2 with high potency. J. Biol. Chem..

[5] Chatterjee B., Thakur S.S. (2022). Remdesivir and Its Combination with Repurposed Drugs as COVID-19 Therapeutics. Front. Immunol..

[6] Kokic G., Hillen H.S., Tegunov D., Dienemann C., Seitz F., Schmitzova J., Farnung L., Siewert A., Höbartner C., Cramer P. (2021). Mechanism of SARS-CoV-2 polymerase stalling by remdesivir. Nat. Commun..

[7] Malin J.J., Suárez I., Priesner V., Fätkenheuer G., Rybniker J. (2020). Remdesivir against COVID-19 and Other Viral Diseases. Clin. Microbiol. Rev..

[8] Beigel J.H., Tomashek K.M., Dodd L.E., Mehta A.K., Zingman B.S., Kalil A.C., Hohmann E., Chu H.Y., Luetkemeyer A., Kline S. (2020). Remdesivir for the Treatment of COVID-19—Final Report. N. Engl. J. Med..

[9] WHO Solidarity Trial Consortium (2022). Remdesivir and three other drugs for hospitalised patients with COVID-19: Final results of the WHO Solidarity randomised trial and updated meta-analyses. Lancet.

[10] Ali K., Azher T., Baqi M., Binnie A., Borgia S., Carrier F.M., Cavayas Y.A., Chagnon N., Cheng M.P., Conly J. (2022). Remdesivir for the treatment of patients in hospital with COVID-19 in Canada: A randomized controlled trial. Can. Med. Assoc. J..

[11] European Respiratory Journal. https://erj.ersjournals.com/content/57/4/2100048.

[12] Infectious Diseases Society of America. https://www.idsociety.org/practice-guideline/covid-19-guideline-treatment-and-management.

[13] National Institutes of Health. https://www.covid19treatmentguidelines.nih.gov/management/clinical-management-of-adults/hospitalized-adults--therapeutic-management.

[14] Hagman K., Hedenstierna M., Gille-Johnson P., Hammas B., Grabbe M., Dillner J., Ursing J. (2021). Severe Acute Respiratory Syndrome Coronavirus 2 RNA in Serum as Predictor of Severe Outcome in Coronavirus Disease 2019: A Retrospective Cohort Study. Clin. Infect. Dis..

[15] Hogan C.A., Stevens B.A., Sahoo M.K., Huang C., Garamani N., Gombar S., Yamamoto F., Murugesan K., Kurzer J., Zehnder J. (2021). High Frequency of SARS-CoV-2 RNAemia and Association with Severe Disease. Clin. Infect. Dis..

[16] Wong C.K.H., Lau K.T.K., Au I.C.H., Xiong X., Chung M.S.H., Lau E.H.Y., Cowling B.J. (2022). Optimal Timing of Remdesivir Initiation in Hospitalized Patients with Coronavirus Disease 2019 (COVID-19) Administered with Dexamethasone. Clin. Infect. Dis..

[17] Spinner C.D., Gottlieb R.L., Criner G.J., Arribas López J.R., Cattelan A.M., Soriano Viladomiu A., Ogbuagu O., Malhotra P., Mullane K.M., Castagna A. (2020). Effect of Remdesivir vs Standard Care on Clinical Status at 11 Days in Patients with Moderate COVID-19: A Randomized Clinical Trial. JAMA.

[18] Gottlieb R.L., Vaca C.E., Paredes R., Mera J., Webb B.J., Perez G., Oguchi G., Ryan P., Nielsen B.U., Brown M. (2022). Early Remdesivir to Prevent Progression to Severe COVID-19 in Outpatients. N. Engl. J. Med..

[19] European Medicines Agency. https://www.ema.europa.eu/en/medicines/human/EPAR/veklury.

[20] Ader F., Bouscambert-Duchamp M., Hites M., Peiffer-Smadja N., Poissy J., Belhadi D., Diallo A., Lê M.P., Peytavin G., Staub T. (2022). Remdesivir plus standard of care versus standard of care alone for the treatment of patients admitted to hospital with COVID-19 (DisCoVeRy): A phase 3, randomised, controlled, open-label trial. Lancet Infect. Dis..

[21] Pan H., Peto R., Henao-Restrepo A.M., Preziosi M.P., Sathiyamoorthy V., Abdool Karim Q., Alejandria M.M., Hernández García C., Kieny M.P., WHO Solidarity Trial Consortium (2021). Repurposed Antiviral Drugs for COVID-19—Interim WHO Solidarity Trial Results. N. Engl. J. Med..

[22] Garibaldi B.T., Wang K., Robinson M.L., Betz J., Caleb Alexander G., Andersen K.M., Joseph C.S., Mehta H.B., Korwek K., Sands K.E. (2022). Real-World Effectiveness of Remdesivir in Adults Hospitalized with Coronavirus Disease 2019 (COVID-19): A Retrospective, Multicenter Comparative Effectiveness Study. Clin. Infect. Dis..

[23] Olender S.A., Walunas T.L., Martinez E., Perez K.K., Castagna A., Wang S., Kurbegov D., Goyal P., Ripamonti D., Balani B. (2021). Remdesivir Versus Standard-of-Care for Severe Coronavirus Disease 2019 Infection: An Analysis of 28-Day Mortality. Open Forum Infect. Dis..

[24] Benfield T., Bodilsen J., Brieghel C., Harboe Z.B., Helleberg M., Holm C., Israelsen S.B., Jensen J., Jensen T.Ø., Johansen I.S. (2021). Improved Survival Among Hospitalized Patients with Coronavirus Disease 2019 (COVID-19) Treated with Remdesivir and Dexamethasone. A Nationwide Population-Based Cohort Study. Clin. Infect. Dis..

[25] Joo E.J., Ko J.H., Kim S.E., Kang S.J., Baek J.H., Heo E.Y., Shi H.J., Eom J.S., Choe P.G., Bae S. (2021). Clinical and Virologic Effectiveness of Remdesivir Treatment for Severe Coronavirus Disease 2019 (COVID-19) in Korea: A Nationwide Multicenter Retrospective Cohort Study. J. Korean Med. Sci..

[26] Arch B., Kovacs D., Scott J.T., Jones A.P., Harrison E.M., Rosala-Hallas A., Gamble C.G., Openshaw P.J.M., Baillie J.K., Semple M.G., on behalf of ISARIC4C Investigators (2021). Evaluation of the effectiveness of remdesivir in treating severe COVID-19 using data from the ISARIC WHO Clinical Characterisation Protocol UK: A prospective, national cohort study. medRxiv.

[27] Mozaffari E., Chandak A., Zhang Z., Liang S., Thrun M., Gottlieb R.L., Kuritzkes D.R., Sax P.E., Wohl D.A., Casciano R. (2022). Remdesivir Treatment in Hospitalized Patients with Coronavirus Disease 2019 (COVID-19): A Comparative Analysis of In-hospital All-cause Mortality in a Large Multicenter Observational Cohort. Clin. Infect. Dis..

[28] Garcia-Vidal C., Meira F., Cózar-Llistó A., Dueñas G., Puerta-Alcalde P., Garcia-Pouton N., Chumbita M., Cardozo C., Hernandez-Meneses M., Alonso-Navarro R. (2021). Real-life use of remdesivir in hospitalized patients with COVID-19. Rev. Esp. Quimioter..

[29] Garcia-Vidal C., Alonso R., Camon A.M., Cardozo C., Albiach L., Agüero D., Marcos M.A., Ambrosioni J., Bodro M., Chumbita M. (2021). Impact of remdesivir according to the pre-admission symptom duration in patients with COVID-19. J. Antimicrob. Chemother..

[30] Paranjape N., Husain M., Priestley J., Koonjah Y., Watts C., Havlik J. (2021). Early Use of Remdesivir in Patients Hospitalized With COVID-19 Improves Clinical Outcomes: A Retrospective Observational Study. Infect. Dis. Clin. Pract..

[31] French G., Hulse M., Nguyen D., Sobotka K., Webster K., Corman J., Aboagye-Nyame B., Dion M., Johnson M., Zalinger B. (2022). Impact of hospital strain on excess deaths during the COVID-19 pandemic-United States, July 2020–July 2021. Am. J. Transplant..

[32] Wikipedia. https://en.wikipedia.org/wiki/COVID-19_pandemic_in_Greece.

[33] National Public Health Organization. https://eody.gov.gr/neos-koronaios-covid-19/.

[34] Hellenic Society for Infectious Diseases. https://www.loimoxeis.gr/covid-19-info-banner/.

